# Supplementary material for: Overarching Principles and Dimensions of the Functional Organization in the Inferior Parietal Cortex
Source: Cereb Cortex. 2020 Jun 9;30(11):5639–53. doi: 10.1093/cercor/bhaa133 (PMC7116231; doi:10.1093/cercor/bhaa133)
Supplement: Supplementary_bhaa133 [file supplementary_bhaa133.docx]

Supplementary

Supplementary Table 1. The locations of the activation peaks from the GLM analysis.

|  | cluster size | T | x | y | z | Location |
| --- | --- | --- | --- | --- | --- | --- |
| Sentences:  Violation > normal | 920 | 9.15 | -42 | 21 | 21 | Inferior frontal Gyrus, BA44 |
|  |  | 9.13 | -48 | 18 | 15 |  |
|  |  | 6.59 | -39 | 3 | 33 |  |
|  | 505 | 7.58 | -51 | -54 | 15 | Angular gyrus |
|  |  | 5.19 | -33 | -42 | -21 | Temporal fusiform |
|  |  | 5.04 | -63 | -51 | 3 | Middle temporal gyrus |
|  | 184 | 5.73 | -30 | -69 | 45 | Angular gyrus, PGa |
|  |  | 5.73 | -27 | -75 | 39 | Superior parietal lobule |
|  | 66 | 5.37 | -12 | -33 | 69 | Postcentral gyrus |
|  | 191 | 5.16 | 48 | -48 | 9 | Angular gyrus, PGa |
|  |  | 5.10 | 51 | -30 | 0 | Superior temporal gyrus |
|  |  | 4.52 | 57 | -42 | 6 | Middle temporal gyrus |
|  | 122 | 4.97 | 48 | 3 | 30 | Precentral gyrus |
|  |  | 4.61 | 48 | 15 | 21 | Inferior frontal Gyrus, BA44 |
|  | 39 | 4.71 | -9 | 18 | 45 | Superior frontal gyrus |
|  | 25 | 4.40 | -36 | -69 | 18 | Angular gyrus, PGp |
|  | 24 | 4.38 | 33 | -66 | 45 | Superior parietal lobule |
| Numbers:  Violation > normal | 84 | 5.08 | -39 | -75 | -3 | Lateral occipital cortex |
|  |  | 3.77 | -27 | -90 | -6 | Occipital pole |
|  | 32 | 4.76 | -12 | -36 | -36 | Cerebellum |
|  |  | 4.06 | 0 | -33 | -39 |  |
|  |  | 4.00 | 12 | -30 | -36 |  |
|  | 81 | 4.42 | -36 | -78 | 24 | Angular gyrus, PGp |
|  |  | 4.08 | -24 | -78 | 24 |  |
| Pictures:  Violation > normal | 2709 | 7.09 | 24 | -57 | -12 | Fusiform gyrus |
|  |  | 6.79 | 33 | -75 | -15 |  |
|  |  | 6.69 | 18 | -78 | -18 |  |
|  |  | 6.65 | -24 | -81 | -15 | Fusiform gyrus |
|  |  | 6.56 | -36 | -84 | -18 | Lateral occipital cortex |
|  |  | 5.95 | -9 | -81 | -6 | Lingual gyrus |
|  |  | 6.40 | -18 | -72 | 48 | Superior parietal lobule, 7P |
|  |  | 5.85 | -27 | -81 | 18 | Angular gyrus, PGp |
|  | 170 | 6.72 | 3 | -42 | 15 | Posterior cingulate |
|  |  | 5.49 | -3 | -24 | 27 | Posterior cingulate |
|  | 111 | 5.93 | 45 | 24 | 39 | Middle frontal gyrus |
|  | 65 | 5.83 | 51 | -51 | 33 | Angular gyrus, PGa |
|  |  | 3.74 | 51 | -51 | 45 |  |
|  | 81 | 4.86 | -39 | 18 | 45 | Inferior frontal gyrus, BA44 |
|  | 19 | 4.44 | -51 | -57 | 30 | Angular gyrus, PGa |
|  | 23 | 4.19 | 33 | 60 | -6 | Frontal pole |
|  |  | 3.68 | 45 | 51 | -6 |  |
| Violation effect: Sentences > numbers & pictures | 75 | 4.33 | -36 | -3 | 33 | Precentral gyrus |
|  |  | 4.26 | -39 | 0 | 51 |  |
|  | 41 | 4.28 | -39 | 18 | 21 | Inferior frontal gyrus, BA44 |
|  | 45 | 4.15 | -54 | -51 | 21 | Angular gyrus, PGa |
|  | 30 | 3.86 | 54 | -30 | 0 | Superior temporal gyrus |
|  |  | 3.84 | 54 | -36 | 6 |  |
| Violation effect: Pictures > sentences & numbers | 102 | 4.70 | 30 | -63 | -12 | Fusiform gyrus |
|  |  | 4.60 | 30 | -78 | -9 |  |
|  |  | 3.82 | 15 | -81 | -12 |  |
|  | 44 | 4.40 | -18 | -81 | -12 | Fusiform gyrus |
|  | 51 | 3.89 | 12 | -90 | 18 | Visual cortex |


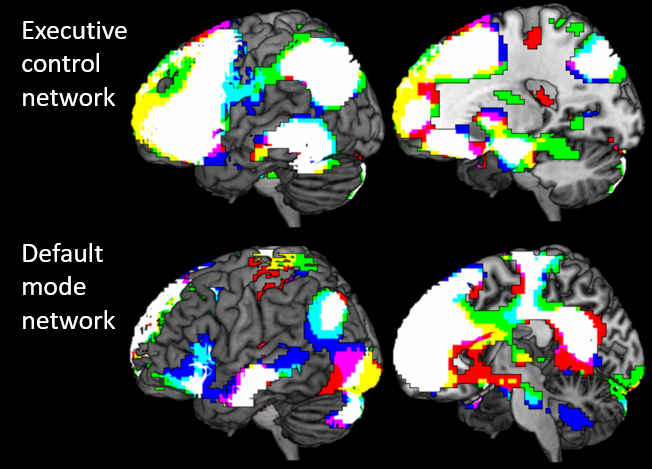


Supplementary Figure 1. The executive network and default mode network for each task: numbers (red), picture (blue), and sentences (green). The overlap between networks is shown in white.


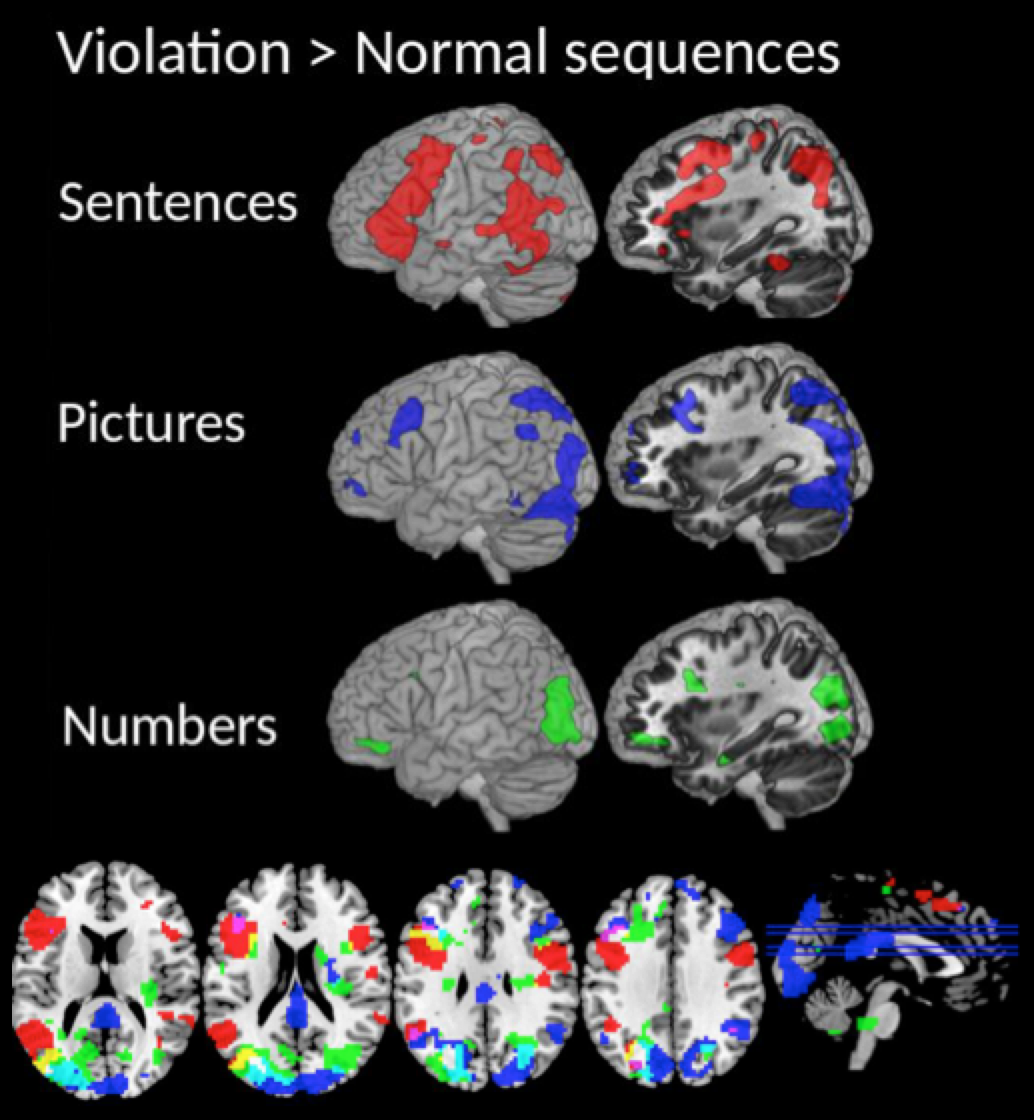


Supplementary Figure 2. Top panel: The results from the GLM analysis for the Violation > normal sequence contrast for each task (uncorrected, p < .001 for visual purposes). Bottom panel: axial slices showing the activation from each task. Yellow = overlap between sentences and numbers, Cyan = overlap of pictures and numbers, Pink = overlap between sentences and pictures, White = full overlap.


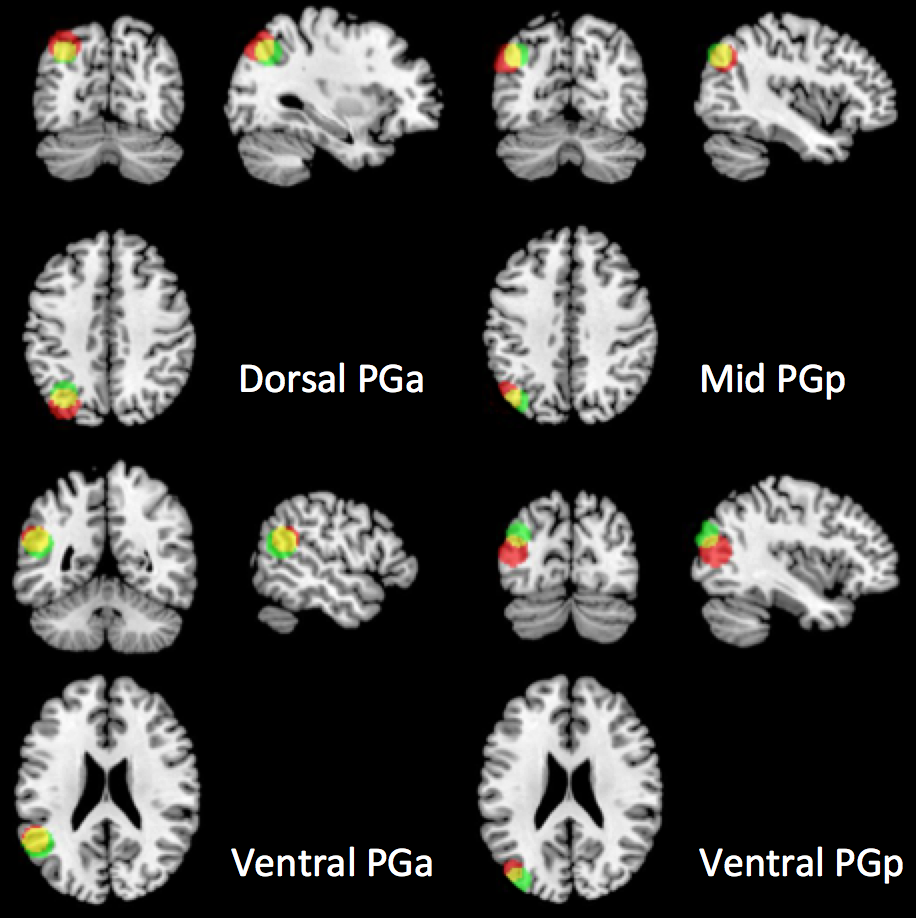


Supplementary Figure 3. The angular gyrus regions of interest (ROIs) derived from the task-ICA (red) and resting-state ICA (green), and the overlap (yellow).
